# Effect of cell permeability and dehydrogenase expression on octane activation by CYP153A6-based whole cell *Escherichia coli* catalysts

**DOI:** 10.1186/s12934-017-0763-0

**Published:** 2017-09-20

**Authors:** Bronwyn E. White, Caryn J. Fenner, Martha S. Smit, Susan T. L. Harrison

**Affiliations:** 1 Centre for Bioprocess Engineering Research (CeBER), Department of Chemical Engineering, University of Cape Town, Private Bag X3, Rondebosch, Cape Town, 7701 South Africa; 2 Department of Microbial, Biochemical and Food Biotechnology, University of the Free State, Bloemfontein, South Africa; 3 South African DST-NRF Centre of Excellence in Catalysis, c*change, University of Cape Town, Private Bag, Rondebosch, Cape Town, 7701 South Africa

**Keywords:** Alkane activation, Octane, CYP153A6, Whole cell biocatalysis, Transport, Membrane permeabilisation, Cofactor regeneration, Glycerol dehydrogenase

## Abstract

**Background:**

The regeneration of cofactors and the supply of alkane substrate are key considerations for the biocatalytic activation of hydrocarbons by cytochrome P450s. This study focused on the biotransformation of n-octane to 1-octanol using resting *Escherichia coli* cells expressing the CYP153A6 operon, which includes the electron transport proteins ferredoxin and ferredoxin reductase. Glycerol dehydrogenase was co-expressed with the CYP153A6 operon to investigate the effects of boosting cofactor regeneration. In order to overcome the alkane supply bottleneck, various chemical and physical approaches to membrane permeabilisation were tested in strains with or without additional dehydrogenase expression.

**Results:**

Dehydrogenase co-expression in whole cells did not improve product formation and reduced the stability of the system at high cell densities. Chemical permeabilisation resulted in initial hydroxylation rates that were up to two times higher than the whole cell system, but severely impacted biocatalyst stability. Mechanical cell breakage led to improved enzyme stability, but additional dehydrogenase expression was necessary to improve product formation. The best-performing system (in terms of final titres) consisted of mechanically ruptured cells expressing additional dehydrogenase. This system had an initial activity of 1.67 ± 0.12 U/g_DCW_ (32% improvement on whole cells) and attained a product concentration of 34.8 ± 1.6 mM after 24 h (22% improvement on whole cells). Furthermore, the system was able to maintain activity when biotransformation was extended to 72 h, resulting in a final product titre of 60.9 ± 1.1 mM.

**Conclusions:**

This study suggests that CYP153A6 in whole cells is limited by coupling efficiencies rather than cofactor supply. However, the most significant limitation in the current system is hydrocarbon transport, with substrate import being the main determinant of hydroxylation rates, and product export playing a key role in system stability.

**Electronic supplementary material:**

The online version of this article (doi:10.1186/s12934-017-0763-0) contains supplementary material, which is available to authorized users.

## Background

In recent decades there has been renewed interest in the activation of alkanes to position these widely available but unreactive hydrocarbons as a potential feedstock for the creation of diverse platform chemicals [1, 2]. Owing to the stable nature of these molecules, chemical activation requires the use of high temperatures or harsh oxidants. Current methods are prone to low regioselectivity and over-activation [1, 3, 4]. To this end, the use of enzymes as biocatalysts has been explored [4, 5]. The enzymes of the cytochrome P450 family have attracted attention owing to their diverse substrate range and high efficiencies for regiospecific terminal oxidation [2, 6, 7]. However, there are still numerous obstacles to be overcome if biocatalytic alkane activation is to be feasible at an industrial scale, including the enzymes’ low activities and multi-component nature, their demand for cofactors, low substrate solubility and high toxicity of substrates and products [8–10].

The supply of cofactors can be a limiting step in hydrocarbon biotransformation, even where in vivo systems are used to provide cellular machinery for endogenous cofactor regeneration [3, 8, 11]. The co-expression of a dehydrogenase alongside the biotransformation enzyme can boost the cell’s capacity to regenerate cofactors [11–14]. There is, however, a risk that the increased metabolic burden may impact negatively on cell physiology and functioning [15, 16].

Limitations in passive transport of hydrocarbon substrates from the organic phase to the enzyme may be a more significant obstacle, as this results in unfavourable ratios between substrate and product in the vicinity of the hydroxylase, leading to slow reaction kinetics and encouraging over-oxidation of product, as supported by Grant et al. [17]. The low solubility and diffusion of hydrocarbons in the aqueous culture medium presents a challenge, but the key bottleneck is transport of hydrocarbons across the cell membrane [17–19]. To overcome this limitation, the membrane can be permeabilised via chemical solubilisation [18, 19], genetic mutation of key lipoproteins [20], or the co-expression of cross-membrane transporter proteins alongside the biotransformation enzyme [18, 21–23]. These techniques for enhanced transport are able to boost activities of biotransformation enzymes, although they often have negative effects on cell physiology and enzyme stability [18, 20, 22].

This study investigated cofactor supply and passive cross-membrane transport of substrate and product, using octane as a model substrate. The system consisted of resting *Escherichia coli* whole cells expressing a heterologous cytochrome P450, CYP153A6, and its natural electron transport partners, ferredoxin reductase and ferredoxin. Additional glycerol dehydrogenase was expressed alongside the CYP153A6 in an attempt to overcome the cofactor bottleneck. In order to investigate the transport bottleneck, membrane permeabilisation was carried out, using either exposure to chemical additives (acetone, Triton X-100 or polymyxin B) or mechanical breakage of cells.

## Results and discussion

### Effects of glycerol dehydrogenase over-expression in whole cells

For the investigation of cofactor effects, whole cells expressing the CYP153A6 operon, including ferredoxin and ferredoxin reductase (abbreviated as CYP), were compared to whole cells expressing CYP as well as additional heterologous glycerol dehydrogenase (abbreviated as CYP + GLD). Experiments were performed for low and high cell density cultures. In low cell density cultures the dehydrogenase was expressed on pCDFDuet, while in high cell density cultures the dehydrogenase was expressed on pACYCDuet. CYP was expressed on pET28b+, and CYP-only strains also carried the appropriate empty Duet vector. The systems converted n-octane into 1-octanol; some octyl acetate by-product was also observed when the pACYCDuet vector was present. Octyl acetate was not observed in whole cell systems expressing pCDFDuet instead of pACYCDuet. The octyl acetate is clearly an artefact of the expression system: pACYCDuet contains a chloramphenicol resistance marker, expressing chloramphenicol acetyltransferase (CAT), which attacks one of the hydroxyl groups present on the chloramphenicol molecule. CAT has been shown to acetylate perillyl alcohol resulting from the hydroxylation of limonene by a cytochrome P450 [24], so it is the likely agent in the acetylation of 1-octanol.

In order to catalyse hydroxylation reactions, the CYP153A6 system requires electrons, which it obtains from the reduced form of the cofactor nicotinamide adenine dinucleotide (NADH). The oxidised form of the cofactor (NAD^+^) must be regenerated or the reaction will quickly exhaust the available cofactor supply. One of the benefits of in vivo systems is that cellular metabolism provides cofactor regeneration [8, 9]. However, spectrophotometric assays performed in the absence of substrate suggested that the system in this study oxidised cofactor as fast as the cells could reduce it (Additional file 1: Figure S1; Table 1). It was hypothesised that cofactor supply was a limiting factor in the biotransformation of octane, and that the bottleneck could be alleviated through the co-expression of additional heterologous glycerol dehydrogenase. This strategy of boosting dehydrogenase expression to improve turnovers has been applied successfully in other hydrocarbon systems; for example, in the co-expression of alcohol dehydrogenase with CYP105A1 for hydroxylation of abietic acid [25], glucose dehydrogenase with an aldehyde reductase from *Sporobolomyces salmonicolor* for reduction of ethyl 4-chloro-3-oxobutanoate [14], glucose dehydrogenase with a P450_BM-3_ mutant for oxyfunctionalisation of α-pinene [13], and glycerol dehydrogenase with P450cam for oxygenation of camphor [12]. In this study, systems co-expressing additional glycerol dehydrogenase were shown to be capable of producing cofactor in excess of the cell’s non-biotransformation usage (Additional file 1: Figure S1; Table 1).

**Table 1:** Glycerol dehydrogenase activities in whole cells

	48 h Incubation without substrate	48 h Biotransformation
CYP	−0.01 ± 0.02	0.00 ± 0.02
CYP + GLD	1.81 ± 0.87	1.49 ± 0.32

Units of activity are µmoles NAD^+^ reduced per minute per mL. The above assays were performed on samples from low cell density cultures. The values displayed represent averages over two biological replicates, with multiple time points sampled per replicate

**Fig. 1:**
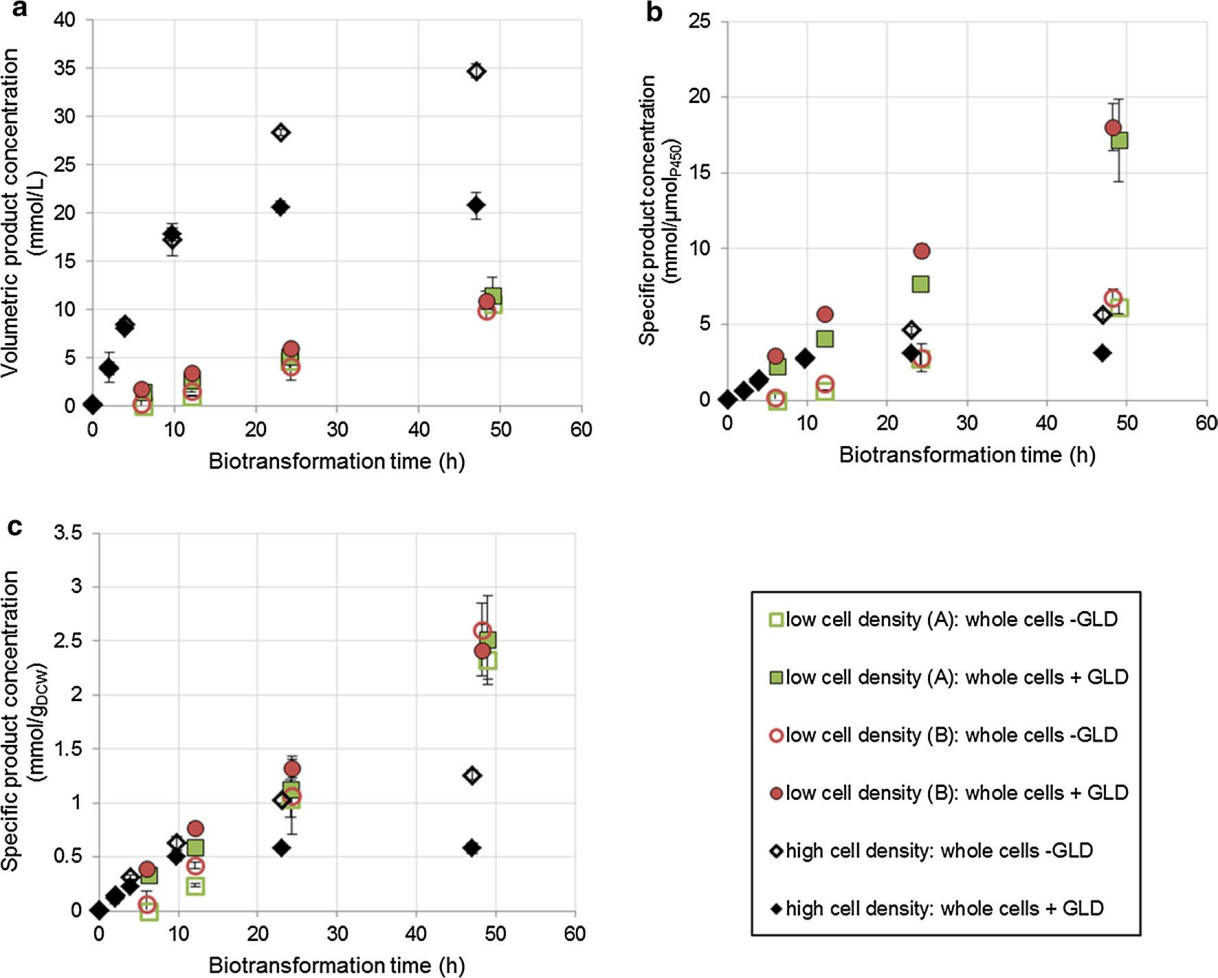
Product formation in whole cells over 48 h of biotransformation. Low cell density was between 3.5 and 5 g_DCW_/L. High cell density was >25 g_DCW_/L. High cell density cultures produced octyl acetate by-product alongside 1-octanol (an artefact of the expression system). The product concentrations shown here are the combined concentrations of 1-octanol and octyl acetate. Two vials were sacrificed to obtain each sample point. The organic phase was extracted into ethyl acetate and analysed via gas chromatography

However in the CYP153A6 systems, when octane was added to whole-cell cultures co-expressing additional dehydrogenase, product titres did not improve relative to cells expressing only the CYP operon (Fig. 1a), even though higher dehydrogenase activity should have been making more cofactor available (Table 1). In low cell density cultures, strains with excess NADH turnover produced a comparable amount of 1-octanol to those with unboosted cofactor regeneration rates (Fig. 1a). Specific production per gram dry cell weight was also not substantially different over the 48 h period (Fig. 1c). While specific production rates in terms of mmol product per µmol active P450 were higher for cells containing extra GLD (Fig. 1b), this was more likely due to reduced expression of CYP153A6 (Table 2). Looking at the CYP strain, low cell density cultures had substantially more active P450 per unit cells than high cell density cultures (0.382 vs 0.224 µmol_P450_/g_DCW_), yet their production per unit cells over the first 10–12 h was slightly lower, suggesting that CYP153A6 concentration was not limiting in the CYP strains. A similar argument can be made for the CYP + GLD strains: the high cell density culture had a slightly higher specific P450 concentration than the low cell density cultures (0.187 vs 0.140 µmol_P450_/g_DCW_), yet it did not show higher rates per cell over the first 10–12 h.

**Table 2:** Average concentrations of active P450 over 24 h of biotransformation

Culture description	Volumetric concentration (µM)	Specific concentration (µmol_P450_/g_DCW_)
Low cell density (A): whole cells −GLD	1.72	0.380 ± 0.013
Low cell density (A): whole cells +GLD	0.67	0.146 ± 0.005
Low cell density (B): whole cells −GLD	1.47	0.384 ± 0.018
Low cell density (B): whole cells +GLD	0.61	0.134 ± 0.006
High cell density: whole cells −GLD	6.16 ± 0.31	0.224 ± 0.013
High cell density: whole cells +GLD	6.68 ± 0.33	0.187 ± 0.014
Rupture −GLD	4.93 ± 0.25	0.280 ± 0.068
Rupture +GLD	4.13 ± 0.28	0.238 ± 0.018
Disintegration −GLD	4.00 ± 0.13	0.247 ± 0.025
Disintegration +GLD	3.77 ± 0.13	0.240 ± 0.008
Cell free extract −GLD	3.91 ± 0.21	0.243 ± 0.018^a^
Cell free extract +GLD	3.52 ± 0.13	0.219 ± 0.014^a^
Acetone +GLD	5.40 ± 0.27	0.136 ± 0.009
Polymyxin B +GLD	5.98 ± 0.49	0.217 ± 0.019
Triton X-100 +GLD	5.14 ± 0.20	0.153 ± 0.004

Measurements were taken at five time points over the first 24 h of biotransformation. Two vials were sacrificed at each time point for sampling. Concentration of active P450 was determined by CO difference spectrometry in a microwell spectrophotometer using 200 μL (total) aqueous phase. The remaining aqueous phase was used to determine cell dry weight via pelleting and drying. In the case of cell free extract, specific values (marked ^a^) were calculated based on the cell dry weights of the homogenised culture from which the CFE was extracted

**Fig. 2:**
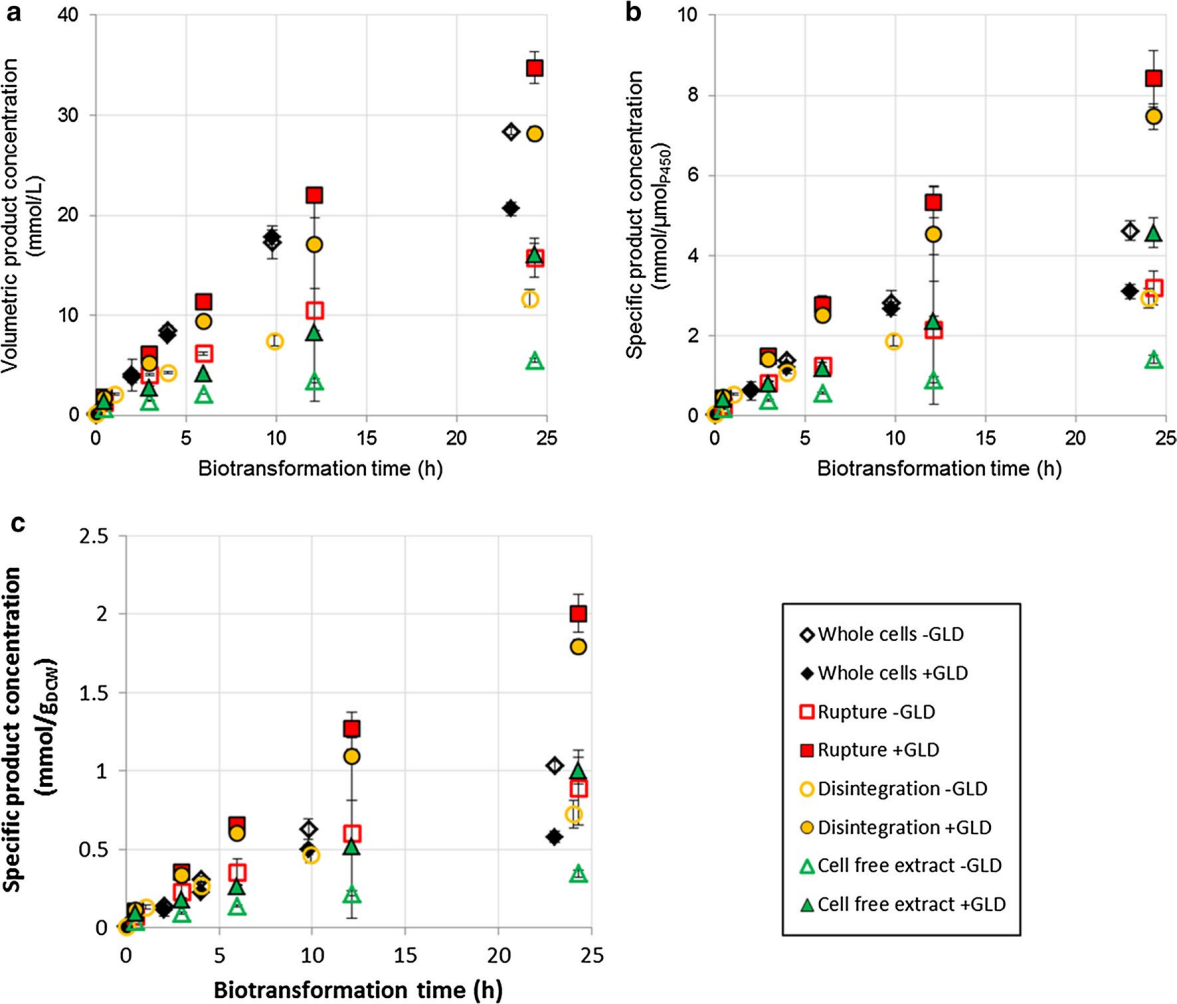
Effect of additional GLD expression on product formation over 24 h, using whole cells, mechanically broken cells or cell free extract. Cultures were at a high cell density, and produced octyl acetate by-product alongside 1-octanol (an artefact of the expression system). The product concentrations shown here are the combined concentrations of 1-octanol and octyl acetate. Two vials were sacrificed to obtain each sample point. The organic phase was extracted into ethyl acetate and analysed via gas chromatography

In high cell density cultures, both strains showed similar volumetric titres for the first 10 h of biotransformation, after which the productivity of the CYP + GLD strain fell off sharply (Fig. 1a). Again, this effect was also visible in the cell-based specific product concentration (Fig. 1c) and was slightly reduced in the P450-based specific product concentration owing to lower P450 expression in the CYP + GLD strain (Fig. 1b). Even in the high density CYP culture, cell-specific activity began lagging visibly after 10 h. In contrast, low density cultures were still maintaining their initial activities after 48 h of biotransformation. This suggests a volumetric limitation, which is exacerbated by an excess of cofactor. Product toxicity would be the most obvious culprit, but since it was later found that mechanically permeabilised cells expressing additional GLD were able to maintain their activity relatively well at product concentrations exceeding those generated by whole cells (Fig. 2), it is clearly not the absolute volume of product that matters. Also, product toxicity cannot explain why whole cells expressing additional dehydrogenase matched the volumetric production of whole cells for the first 10 h, but thereafter lost activity more rapidly. A more plausible explanation would be uncoupling of the hydroxylation reaction. Amongst the uncoupling reactions of P450 systems are the production of hydrogen peroxide (H_2_O_2_) and the production of superoxide, which goes on to form H_2_O_2_ [26]. An excess of H_2_O_2_ is known to be detrimental to cellular functioning [27]. This could explain why the inhibition appears at high cell loadings (which correspond to higher volumetric enzyme concentrations) as well as why the presence of additional dehydrogenase causes the problem to worsen—the excess NADH is channelled into harmful uncoupling reactions instead of being used for hydroxylation of octane. This argument is further supported by the NADH turnovers in biotransforming cells, which are comparable to those in non-biotransforming cells (Table 1). In other words, the P450 enzyme was not limited by cofactor supply, at least in whole cells—rather, it was failing to access its preferred substrate, so it converted oxygen into superoxides or H_2_O_2_ instead.

### Effects of mechanical cell breakage

Alkanes are sparingly soluble in water and do not easily pass through the cell membrane, and hence the supply of substrate has been identified as a major bottleneck in enzymatic hydrocarbon activation [27]. Some literature suggests that the key step is transfer of hydrocarbons across the outer membrane, and that altering the membrane structure to make it more permeable can improve hydroxylation of these substrates. Mutation of the Braun’s lipoprotein to disrupt membrane rigidity improved toluene dioxygenase reaction rates by up to six times [20], while the expression of “channel” proteins allowing for passive diffusion of aromatics and terpenes increased product concentrations by up to five times [23]. To investigate the extent to which cross-membrane transport hindered the octane bioconversion, cells were homogenised at 15 kpsi (enough to rupture the cell membranes) and 30 kpsi (enough to disintegrate the cell membranes). Treated cells were compared to whole cells and cell free extracts. High cell density cultures of cells carrying the pACYCDuet plasmid were used.

Over 24 h, CYP + GLD strains where cells were ruptured or disintegrated managed to match or exceed the titres generated by whole cells (Fig. 2a) despite significantly lower volumetric P450 concentrations (Table 2). As a result, the permeabilised cells outperformed the whole cells in terms of specific productivities (Fig. 2b, c). Overcoming the transport limitation was essential for boosting effectiveness of the biocatalytic enzyme. This is in line with numerous other studies [18, 20, 22, 23, 25, 28, 29]. However, mechanically permeabilised cells that were not expressing additional GLD did not perform well relative to whole cells. Over a 24 h period they produced significantly lower volumetric titres, as well as demonstrating lower hydroxylation rates per unit enzyme and per unit cells. This suggests that some factor other than substrate transport was limiting in the CYP strains. The specific P450 concentrations of the permeabilised cells were higher than those that were previously argued to be non-limiting (Table 2), hence a lack of catalytic enzyme is unlikely to have been the cause. It is possible that the lack of an intact membrane resulted in cofactors “leaking out” and being distributed throughout the aqueous phase, meaning mechanically permeabilised cells had reduced access to cofactors. This would explain why only CYP + GLD strains exhibited improved production rates: the additional dehydrogenase expression was necessary to compensate for the dehydrogenase and cofactors lost due to permeabilisation. It could also explain why the CYP + GLD permeabilised cells did not experience the “poisoning” effect evident in CYP + GLD whole cells, where activity dropped off sharply after 10–12 h. Since permeabilised cells did not have an excess of cofactor, they did not experience excessive uncoupling and hence were not exposed to the same levels of H_2_O_2_.

It is unsurprising that cell free extract substantially underperformed relative to disintegrated cells and whole cells (with or without additional GLD expression), since cells are known to provide some benefit to enzyme functioning by maintaining a stable environment and ensuring proximity of interacting species [27]. However, the P450 in cell free extract retained its activity remarkably well (Table 3), such that CYP + GLD cell free extract was able to surpass the performance of CYP whole cells on a specific basis, when biotransformation time was extended (Additional file 2: Figure S2). However, productivity remained well below that of the ruptured or disintegrated cells.

**Table 3:** Change in product formation rates over 24 h of biotransformation

Culture description	Initial rate^a^ (mmol L^−1^ min^−1^)	Final rate^b^ (mmol L^−1^ min^−1^)	Rate loss over 24 h (%)
Whole cells −GLD	0.0348 ± 0.0020	0.0140 ± 0.0017	60
Whole cells +GLD	0.0325 ± 0.0010	0.0036 ± 0.0001	89
Rupture −GLD	0.0185 ± 0.0001	0.0071 ± 0.0099	62
Rupture +GLD	0.0290 ± 0.0003	0.0175 ± 0.0015	40
Disintegration −GLD	0.0131 ± 0.0007	0.0058 ± 0.0006	56
Disintegration +GLD	0.0232 ± 0.0013	0.0151 ± 0.0050	35
Cell free extract −GLD	0.0054 ± 0.0003	0.0028 ± 0.0001	48
Cell free extract +GLD	0.0089 ± 0.0009	0.0106 ± 0.0013	0
Acetone +GLD	0.0187 ± 0.0010	0.0084 ± 0.0011	55
Polymyxin B +GLD	0.0626 ± 0.0009	0.0034 ± 0.0016	95
Triton X-100 +GLD	0.1060 ± 0.0015	0.0002 ± 0.0006	100

Measurements were taken at five time points over the first 24 h of biotransformation. Two vials were sacrificed at each time point for sampling. The organic phase was extracted into ethyl acetate and analysed via gas chromatography. Rates are based on the combined concentrations of 1-octanol and octyl acetate, which were the only significant products observed

^a^Calculated between 0 and 4 h of biotransformation

^b^Calculated between 10 and 24 h of biotransformation

It is worth noting that the best initial activity achieved by the CYP153A6 resting systems was 2.02 ± 0.16 U/g_DCW_ (disintegration +GLD). This is well below the activities that *E. coli* have been shown to support. For example, Favre-Bulle et al. showed that growing *E. coli* expressing the alkane hydroxylase system of *Pseudomonas oleovorans* were able to hydroxylate n-octane at rates of up to 15 U/g_DCW_ [30]. Resting *E. coli* can achieve similar rates—at low cell densities, Olaofe et al. observed hydroxylation rates of up to 16 U/g_DCW_ 1-octanol for the CYP153A6 system [10]. If higher biocatalyst loading results in reduced specific activity, it suggests an as-yet unidentified physiological limitation.

### Chemical permeabilisation in the presence of additional dehydrogenase

Aside from cell homogenisation, it is possible to permeabilise the membrane through the addition of certain chemicals. This study considered acetone, which increases membrane fluidity by disrupting lipid packing [31]; polymyxin B, an antibiotic peptide which partitions into the outer membrane and disrupts its structure [32]; and Triton X-100, a surfactant which has also been shown to disrupt *E. coli* outer membranes [33]. Since mechanically permeabilised cells had performed so poorly in the absence of additional dehydrogenase, chemical and mechanical permeabilisers were compared using CYP + GLD systems. However, the whole cell system included for comparison did not contain the additional dehydrogenase gene, given that CYP + GLD strains had underperformed when not permeabilised. High cell density cultures carrying the pACYCDuet plasmid were used.

**Fig. 3:**
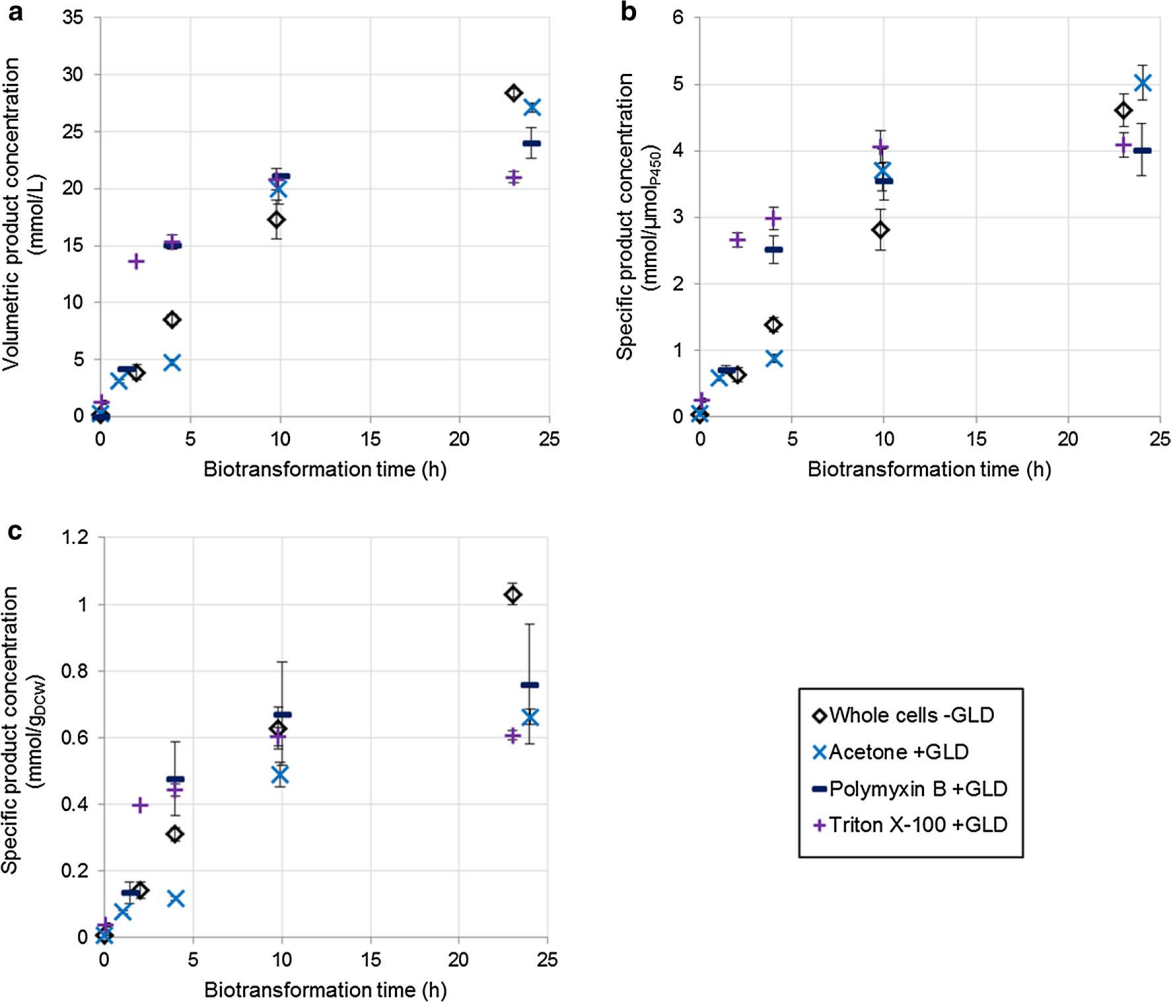
Effect of additional GLD expression on product formation over 24 h, using chemically permeabilised cells expressing additional GLD. Whole cells without additional GLD are included for comparison. Cultures were at a high cell density, and produced octyl acetate by-product alongside 1-octanol (an artefact of the expression system). The product concentrations shown here are the combined concentrations of 1-octanol and octyl acetate. Two vials were sacrificed to obtain each sample point. The organic phase was extracted into ethyl acetate and analysed via gas chromatography

**Fig. 4:**
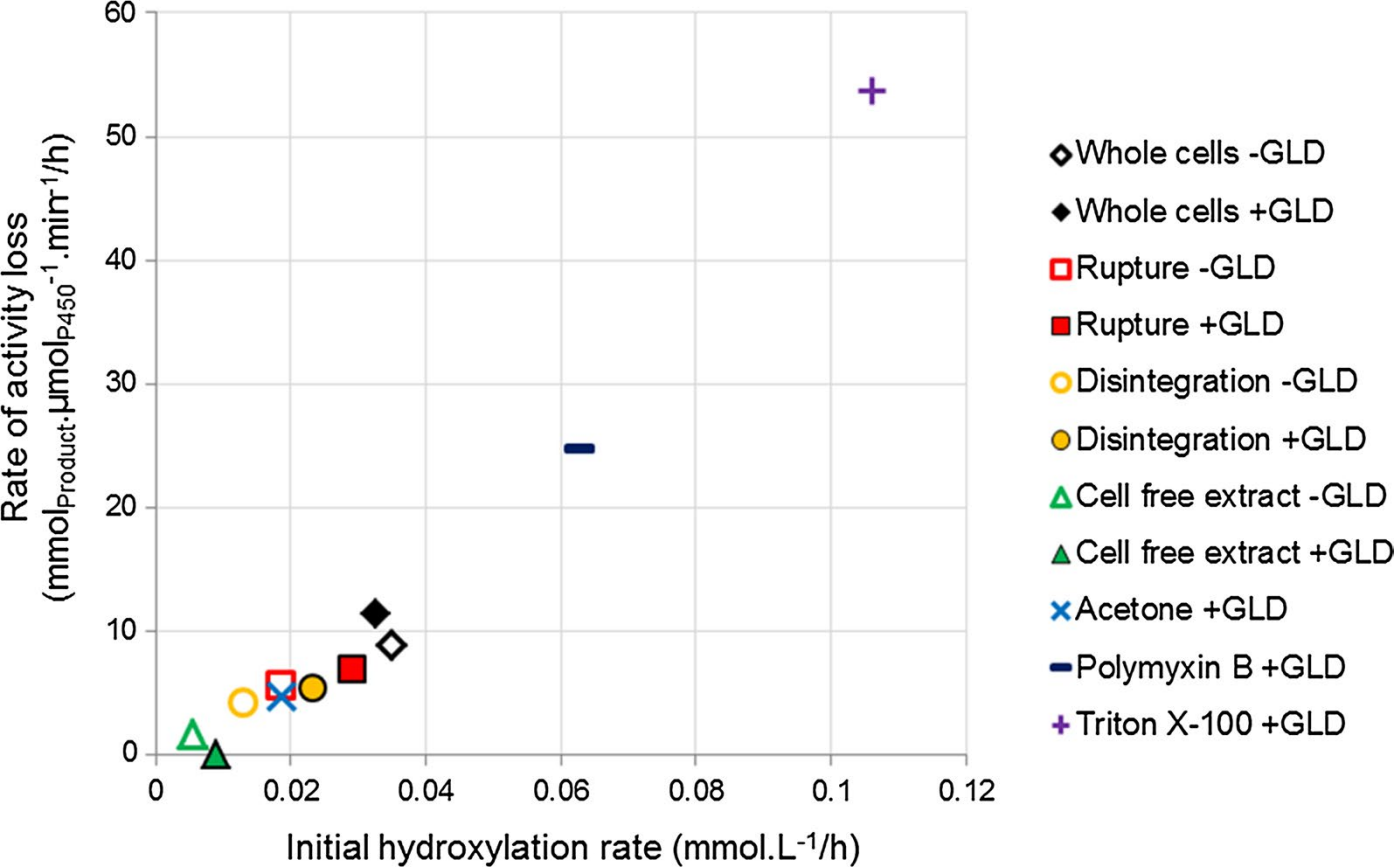
Loss of P450 enzyme performance in various high cell density systems, as a function of initial hydroxylation rate. Values show a good linear correlation (R^2^ = 0.97)

The presence of Triton X-100 or polymyxin B had a positive effect on the initial hydroxylation rates (Fig. 3), which were higher than for any other system on both a volumetric and a specific basis. Initial volumetric rates were two to three times higher for these chemical permeabilisers than for whole cell CYP cultures (Table 3). However, after the first few hours of biotransformation the chemically permeabilised systems exhibited rapid activity loss, with minimal product formation after 10 h, resulting in final titres up to 35% lower than those of whole cell CYP cultures and up to 65% lower than those of ruptured CYP + GLD cultures. As in other experiments, the same trends were present when considering production per unit enzyme, but the effects were dampened because of the reduced P450 concentration in the chemically permeabilised strains. These results are in agreement with authors such as Janocha and Bernhardt (2013) who found that polymyxin B could greatly enhance biotransformation rates, and that the effects were maximal when cofactor regeneration was boosted through heterologous expression. However, these authors used a limiting concentration of substrate and therefore did not investigate whether the high initial rates could be maintained. Considered over extended periods, the use of Triton X-100 has led to mixed results in the literature, with Grant et al. reporting a positive effect on the oxidation of dodecane in microwells and shake flasks when Triton X-100 was present [19], while Julsing et al. found that Triton X-100 was not helpful in the oxidation of fatty acid methyl esters [18]. Bordeaux et al. found that Triton X-100 was moderately helpful in octane bioconversions when the organic phase included a co-solvent, but that the surfactant suppressed turnovers considerably when the organic phase consisted of pure octane [34]. Julsing et al. also used pure substrate as the organic phase, while Grant et al. had dimethylsulfoxide (DMSO) present in the organic phase, and found that the beneficial effects of Triton X-100 were enhanced when more DMSO was present. This suggests that the effectiveness of Triton X-100 is somehow linked to in situ product extraction. This study demonstrated a linear inverse correlation between initial hydroxylation rates and ability of P450 enzymes to maintain hydroxylation activity over 24 h of biotransformation (Fig. 4). The loss of activity was not linked to a loss of active P450, which did not decrease significantly over 24 h (Additional file 3: Figure S3). It also could not have been due to overall product concentration, since mechanically permeabilised cells expressing additional dehydrogenase had significantly higher final rates despite achieving higher final titres (Table 3; Figs. 2, 3). It is therefore hypothesised that it is the intracellular octanol concentration, rather than the volumetric product concentration, which results in negative toxicity effects. Systems permeabilised with polymyxin B or Triton X-100 produced 1-octanol more rapidly than it could be exported from the cell, leading to rapid intracellular build-up of toxic product which had a negative effect on cell physiology, hindering the bioconversion. Mechanically permeabilised cells, or cell free extract, where the product was produced less rapidly and could more easily move out of contact with cellular components, maintained their biocatalytic activities best (Table 3). This effect was not observed in ruptured or disintegrated cells that were not expressing additional dehydrogenase.

Acetone-treated cells performed poorly. Although they were able to reach the same titres as whole cell CYP cultures and performed comparably per unit enzyme (Fig. 3a, b), measurements of cell dry weight confirmed that cell density in the acetone experiment had been higher, while P450 concentration was slightly lower (Table 2). Per unit dry cell weight, acetone-treated cells showed lower initial rates than whole cells (Fig. 3c) with a comparable loss of activity over 24 h (Table 3), reducing the amount of product per cell by almost one-third.

### Passive transport in relation to by-product formation

**Fig. 5:**
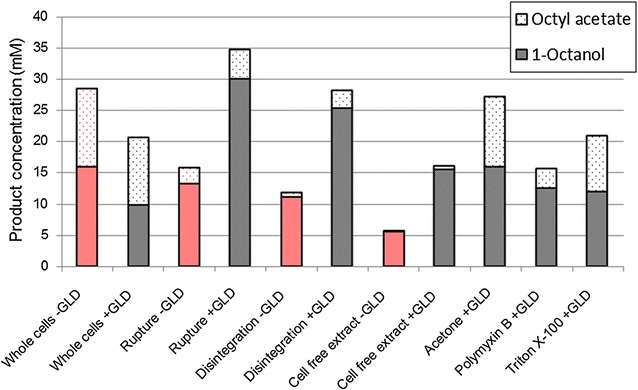
Comparison of 1-octanol product and octyl acetate by-product formed in various high cell density systems over 24 h biotransformations. Two vials were sacrificed to obtain each sample point. The organic phase was extracted into ethyl acetate and analysed via gas chromatography

Although octyl acetate is an artefact of the pACYCDuet expression system, it provides an indication of how easily 1-octanol can exit the cell, since the extent of the acetylation reaction will be greater in cases where the 1-octanol produced by the P450 remains intracellular for longer periods. Grant et al. previously noted that changing phase ratios to improve substrate access and product extraction reduced overoxidation of dodecane in whole cell hydroxylation systems [19]. Comparing the concentration of 1-octanol and octyl acetate after 24 h of biotransformation, it can be seen that mechanically breaking the membrane greatly reduced the extent of by-product formation, as did treatment with polymyxin B (Fig. 5). The more compromised the membrane integrity, the lower the by-product formation. Triton X-100 did not have any significant effect relative to whole cells, possibly because it acts more as a hydrocarbon solubiliser than a membrane permeabiliser, and hence did not enhance product export to the same degree as substrate import. Acetone treatment was also ineffective at reducing by-product formation. Meanwhile, the expression of additional dehydrogenase in whole cells increased octyl acetate formation. Since this cannot be due to reduced product export relative to whole cells without GLD, it is possible that this system had higher levels of CAT expression and hence higher acetylation activity.

## Conclusions

The results of this study suggest that the cofactor requirements of CYP153A6 can typically be supplied by in vivo metabolic processes, and that boosting cofactor supply merely encourages harmful uncoupling reactions. The current system limitation is substrate supply; improving the passive transport into the cell through addition of Triton X-100 or polymyxin B led to significant improvements in initial rates of n-octane hydroxylation. However, product export is also an important factor, since the rapid accumulation of 1-octanol within the cell is cytotoxic. This led to cells being unable to maintain high hydroxylation rates for extended periods, and ultimately resulted in lower final titres for chemically permeabilised cells. Mechanically permeabilised cells, which presumably had improved product export, were able to maintain their initial hydroxylation activities for extended periods, allowing them to attain significantly higher production per cell. However, these mechanically permeabilised systems required the expression of additional dehydrogenase to compensate for cofactors lost during preparation. Further it requires an additional process unit operation, adding to process complexity. With this in mind, alternative permeabilisation techniques are worth considering. In recent years there has been much work done on membrane transporter proteins, with Julsing et al. demonstrating the use of AlkL to enhance hydroxylation of fatty acid methyl esters [18], and Scheps et al. and Grant et al. subsequently demonstrating their usefulness in the hydroxylation of dodecanoic acid and C12–C16 alkanes [18, 22, 29]. It would be interesting to see how protein-based transport compares to chemical and mechanical enhancements in the CYP153A6 system, and whether such a system could gain any benefit from heterologous dehydrogenase expression.

## Methods

### Chemicals, bacterial strains and plasmids

Chemicals and antibiotics were obtained from Sigma-Aldrich and Merck. *Escherichia coli* BL21(DE3) was used as the host strain. Vectors used were pET28b(+) containing the CYP153A6 operon from *Mycobacterium* sp. HXN-1500, pACYCDuet or pCDFDuet (without inserts) and pACYCDuet or pCDFDuet containing the *E. coli* glycerol dehydrogenase gene. All strains contained the CYP153A6 operon on pET28b+. Strains referred to as ‘CYP’ also carried one of the empty Duet vectors, while strains referred to as ‘CYP + GLD’ also carried one of the Duet vectors expressing glycerol dehydrogenase.

### Pre-cultures, medium preparation and expression of heterologous proteins

Pre-cultures (5–10 mL) consisting of Luria–Bertani (LB) broth (10 g L^−1^ tryptone, 5 g L^−1^ yeast extract, 5 g L^−1^ NaCl) were inoculated from glycerol stocks (previously maintained below −60 °C). Pre-cultures were supplemented with 30 μg mL^−1^ kanamycin and either 30 μg mL^−1^ chloramphenicol (for strains containing pACYCDuet) or 100 μg mL^−1^ streptomycin (for strains containing pCDFDuet). Pre-cultures were then grown overnight (≥12 h) at 37 °C with shaking.

Auto-induction (AI) medium was prepared from sterile solutions and had a final composition as follows: 9.2 g L^−1^ tryptone, 4.6 g L^−1^ yeast extract, 5 g L^−1^ glycerol, 0.5 g L^−1^ glucose, 2 g L^−1^ α-lactose for induction of heterologous expression, 3.3 g L^−1^ (NH_4_)_2_SO_4_, 6.8 g L^−1^ KH_2_PO_4_, 7.1 g L^−1^ Na_2_HPO_4_, 0.24 g L^−1^ MgSO_4_, 30 μg mL^−1^ kanamycin, 30 μg mL^−1^ chloramphenicol, 50 μM FeCl_3_·6H_2_O (Saarchem) and 1 mM δ-aminolevulinic acid.

Aliquots of AI medium were transferred to shake flasks (for a final working volume of 200 mL per 1 L flask volume). Cultures were inoculated at 2% (v/v) and incubated at 20 °C and 200 rpm for 48 h.

### Biocatalyst preparation

#### Harvesting

Following growth and enzyme expression, cells were centrifuged at 5000–7000 rpm and ≤4 °C for 10 min. The cell pellets were re-suspended in 200 mM potassium phosphate buffer (pH 8) containing 200 mM glycerol. Low cell density cultures were resuspended to a concentration of ±20 g_wet cell weight_ L^−1^. High cell density cultures were resuspended to a concentration of ±0.4 g_wet cell weight_ mL^−1^, after which different portions of were subjected to a number of treatments designed to decrease the transportation barrier posed by the cell membrane.

#### Whole cells

A portion of high cell density culture was left untreated for use as a control; the cell suspension was diluted to a final concentration of 200 g_WCW_ L^−1^ using 200 mM potassium phosphate buffer (pH 8) containing 200 mM glycerol and 0.5 mM NADH (final concentration 0.25 mM).

#### Mechanical cell breakage

A portion of high cell density culture was homogenised using a one-shot cell disruptor (Constant Systems Ltd). Cells were homogenised at either 15,000 or 30,000 psi, and the resulting suspensions were diluted 1:1 with 200 mM potassium phosphate buffer (pH 8) containing 200 mM glycerol and 0.5 mM NADH (final concentration 0.25 mM). To create cell free extract, a portion of cells was homogenised at 30,000 psi and then centrifuged at 7000 rpm and 4 °C for 30 min; the resulting supernatant was diluted 1:1 with the buffer described above.

#### Chemical permeabilisation

Acetone (5% v/v) was added to a portion of high cell density culture, which were then vortexed for 3 min and centrifuged at 7000 rpm and 4 °C for 10 min. The cell pellet was washed with phosphate buffer, centrifuged again and re-suspended in the same buffer before being diluted as with whole cells.

A portion of high cell density culture was diluted as with whole cells, except that the buffer also contained polymyxin B (final concentration 30 μM).

A portion of high cell density culture was diluted as with whole cells, except that the buffer also contained Triton X-100 (final concentration 0.25% v/v).

### Biotransformation

Biotransformations were carried out in 40 mL capped amber vials at 20 °C and 200 rpm. The total reaction volume per vial was 1.3–1.45 mL with 0.2 mL n-octane per 1 mL aqueous phase. Low cell density cultures also contained 0.1 mL BEHP per 1 mL aqueous phase. At each time point, two vials were sacrificed for sampling. Wherever practicable, vials and samples were kept on ice after being removed from the incubator.

### Analytical methods

#### Quantification of CYP153A6

Determination of active P450 concentration was based on the spectrophotometric assay described by Guengerich et al. [35], modified for the microliter scale. From each vial, 200 μL of aqueous phase was drawn off, diluted 1:1 with 200 mM potassium phosphate buffer (pH 8) containing 200 mM glycerol, mixed with a few grains of sodium dithionite (Na_2_S_2_O_4_), and split equally between two wells of a flat-bottomed, clear, 96-well clip-in spectrophotometry plate (Greiner). One well was then exposed to carbon monoxide for 5–10 min via incubation in a closed container continuously flushed with CO (sample), while the other well was kept on the workbench (blank). A spectrophotometer (Spectramax M2, molecular devices in the case of high cell density experiments) was used to measure the absorbance of each well between 360 and 500 nm at 2 nm intervals. The blank well was used for baseline correction, after which the concentration of active CYP153A6 was calculated according to the following formula:

$$ Active P450 \,\,( {\upmu \text{M}}) = \frac{{\left( {absorbance\; at\; 450\,{\rm nm}} \right) - \left( {absorbance\; at\; 490\,{\rm nm}} \right)}}{0.091} \times 2 $$

#### Determination of glycerol dehydrogenase activity

Determination of GLD activity was adapted from the method of Lin and Magasanik [36]. The assay solution consisted of 0.1 M carbonate, 0.1 M glycerol, 1 mM NAD^+^ and 33 mM ammonium sulphate, at a final pH of 10.0–10.5. 2.9 mL of assay solution was mixed with 100 µL of aqueous phase (appropriately diluted) and a spectrophotometer (Thermo Scientific Genesys 10S UV–Vis) was used to measure the change in optical density at 340 nm over 3 min, blanked against water. The GLD activity, in terms of µmoles NAD^+^ reduced per minute per mL, was calculated as follows:

$$ GLD \;activity \, \left ( \text{{U/min}} \right) = \frac{{rate\,of\,change\; in\; OD_{340\;{\rm nm}} }}{6.22} \, \times \, 30 \, \times \, (dilution\, factor) $$

#### Extraction and analysis of organic phase

The organic phase was extracted into 1 mL ethyl acetate containing 2 mM decanol as an internal standard. Samples were analysed using via gas chromatography (Shimadzu GC-2010 in the case of high cell density experiments; Varian GC-3900 in the case of low cell density experiments) using a FactorFour VF-5 ms column (95% dimethylpolysiloxane, 60 m × 0.32 mm). A significant unexpected peak was noticed in high cell density experiments, and was identified as octyl acetate via GC–MS of selected samples. The concentration of octyl acetate was calculated by comparing the areas of the 1-octanol and octyl acetate peaks.

The run programme on the Shimadzu was as follows: 1 μL injection volume, split ratio of 50, 2.4 mL min^−1^ H_2_, oven temperature held at 60 °C for 9 min, ramped at 25 °C min^−1^ to 180 °C, held for 1.2 min. The run programme on the Varian was as follows: 0.5 μL injection volume, split ratio of 80, 1 mL min^−1^ N_2_, oven temperature held at 60 °C for 3 min, ramped at 30 °C min^−1^ to 180 °C, held for 7 min.

## Additional files

**Additional file 1: Figure S1.** Change in absorbance at 340 nm over the course of a three minute assay (corrected for dilution factors) giving an indication of rates of NADH regeneration. The above assays were performed on whole cell samples from low cell density resting cultures; cells were not in contact with octane at any point. The values displayed represent averages over two biological replicates, with multiple time points sampled per replicate.

**Additional file 2: Figure S2.** Biotransformations extended to 72 h for mechanically broken cells expressing additional GLD. Whole cells without additional GLD are included for comparison. Cultures were at a high cell density, and produced octyl acetate by-product alongside 1-octanol (an artefact of the expression system). The product concentrations shown here are the combined concentrations of 1-octanol and octyl acetate. Two vials were sacrificed to obtain each sample point. The organic phase was extracted into ethyl acetate and analysed via gas chromatography.

**Additional file 3: Figure S3.** Concentration of active P450 in various high cell density systems over 24 h, determined via CO difference spectrophotometry in a microwell spectrophotometer. Two vials were sacrificed for sampling at each time point.

## Abbreviations

AI: auto-induction (refers to growth/expression medium containing tryptone, yeast extract, glucose, glycerol and lactose)
CAT: chloramphenicol acetyl transferase
CO: carbon monoxide
CYP: refers to strains containing pET28b+ with the operon encoding CYP153A6, FdR and Fdx; and an empty Duet vector (either pACYC or pCDF)
CYP + GLD: refers to strains containing pET28b+ with the operon encoding CYP153A6, FdR and Fdx; and either pACYCDuet or pCDFDuet encoding GLD
Fdx: ferredoxin (“helper protein” associated with CYP153A6)
FdR: ferredoxin reductase (“helper protein” associated with CYP153A6)
GLD: glycerol dehydrogenase
NADH: nicotinamide adenine diphosphate (cellular cofactor utilised by CYP153A6)
U: units of hydroxylation activity, in µmol product formed per minute
