# Supplementary figures and images for: Effect of cell permeability and dehydrogenase expression on octane activation by CYP153A6-based whole cell Escherichia coli catalysts
Source: Microb Cell Fact. 2017 Sep 20;16:156. doi: 10.1186/s12934-017-0763-0 (PMC5607502; doi:10.1186/s12934-017-0763-0)

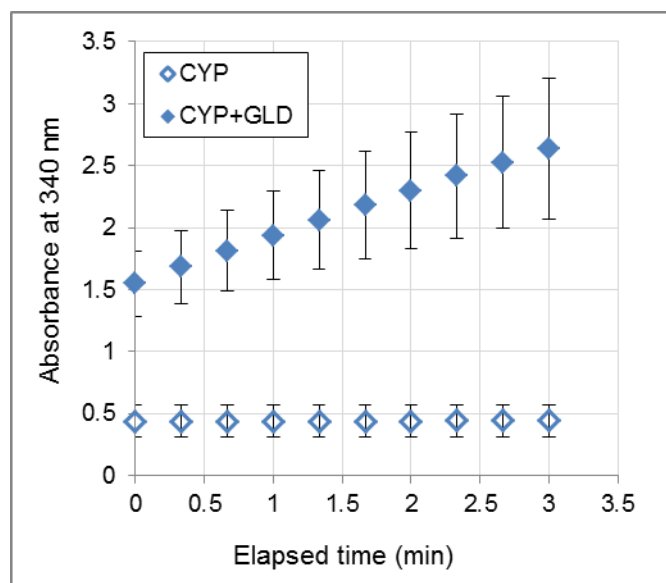

Supplement: Supplementary file 1 — Additional file 1: Figure S1. Change in absorbance at 340 nm over the course of a three minute assay (corrected for dilution factors) giving an indication of rates of NADH regeneration. The above assays were performed on whole cell samples from low cell density resting cultures; cells were not in contact with octane at any point. The values displayed represent averages over two biological replicates, with multiple time points sampled per replicate. [file 12934_2017_763_MOESM1_ESM.pdf]

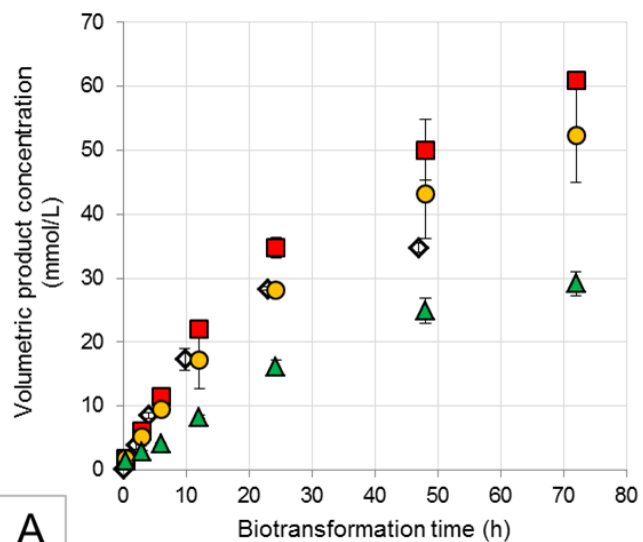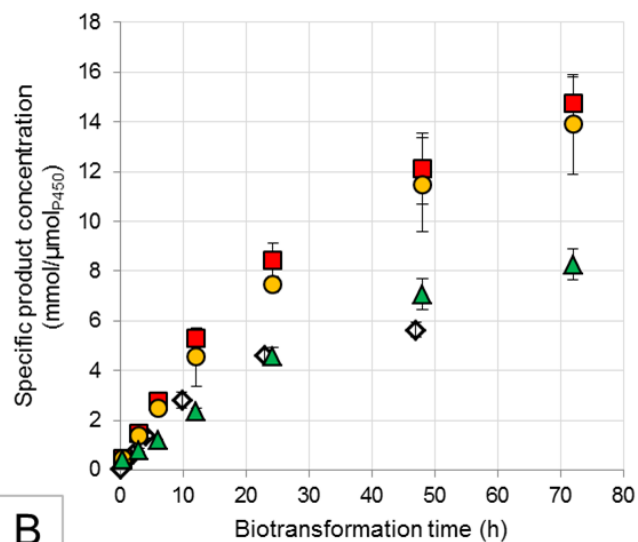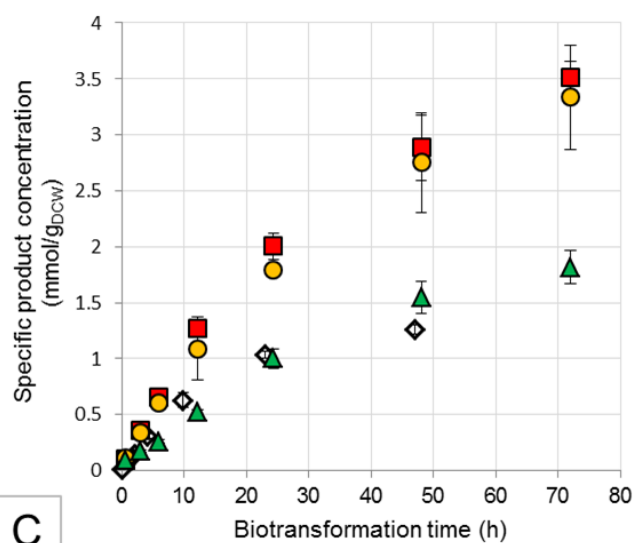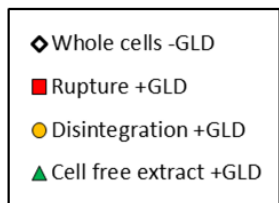

Supplement: Supplementary file 2 — Additional file 2: Figure S2. Biotransformations extended to 72 h for mechanically broken cells expressing additional GLD. Whole cells without additional GLD are included for comparison. Cultures were at a high cell density, and produced octyl acetate by-product alongside 1-octanol (an artefact of the expression system). The product concentrations shown here are the combined concentrations of 1-octanol and octyl acetate. Two vials were sacrificed to obtain each sample point. The organic phase was extracted into ethyl acetate and analysed via gas chromatography. [file 12934_2017_763_MOESM2_ESM.pdf]

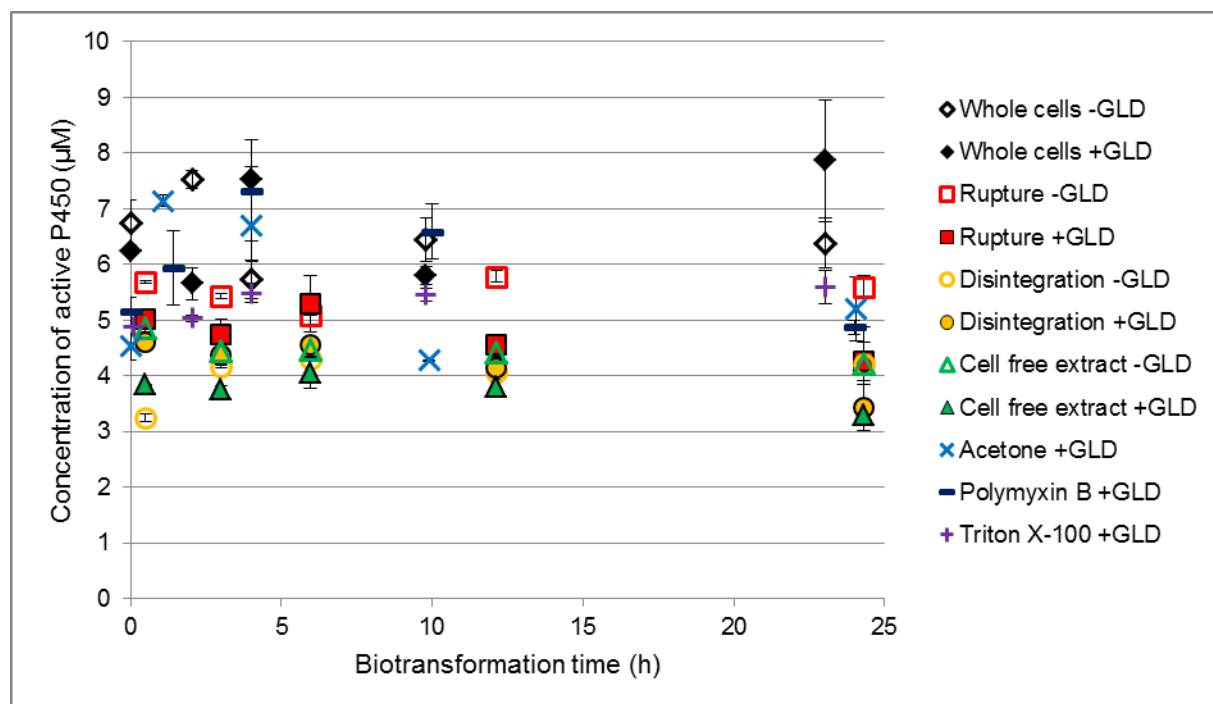

Supplement: Supplementary file 3 — Additional file 3: Figure S3. Concentration of active P450 in various high cell density systems over 24 h, determined via CO difference spectrophotometry in a microwell spectrophotometer. Two vials were sacrificed for sampling at each time point. [file 12934_2017_763_MOESM3_ESM.pdf]
